# Combined treatment with medial unicompartmental knee arthroplasty and anterior cruciate ligament reconstruction is effective on long-term follow-up

**DOI:** 10.1007/s00167-022-07102-3

**Published:** 2022-08-25

**Authors:** Ayham Jaber, Chang min Kim, Alexander Barié, Marcus Streit, Holger Schmitt, Michael Clarius, Christian Merle, Yannic Bangert

**Affiliations:** 1 Department of Orthopedics and Trauma Surgery, Center for Orthopedics, Trauma Surgery and Spinal Cord Injury, Heidelberg University Hospital, Schlierbacher Landstrasse 200a, 69118 Heidelberg, Germany; 2 Department of Orthopedics and Trauma Surgery, GRN-Klinik Schwetzingen, Bodelschwinghstraße 10, 68723 Schwetzingen, Germany; 3 Center for Joint Surgery and Sport Injuries, Sportopaedie Heidelberg, Clinic St. Elisabeth Heidelberg, Max-Reger-Straße 5-7, 69121 Heidelberg, Germany; 4 ARCUS Sportklinik, Pforzheim, Germany; 5 Center for Orthopedics and Sports Trauma Surgery, Atos Clinic Heidelberg, Bismarckstraße 9-15, 69115 Heidelberg, Germany; 6 Vulpius Klinik GmbH, Bad Rappenau, Germany

**Keywords:** Unicompartmental knee arthroplasty, Aanterior cruciate ligament, Anterior cruciate ligament reconstruction, ACL deficiency, Medial osteoarthritis

## Abstract

**Purpose:**

The purpose of the present study was to evaluate the long-term outcome of combined medial unicompartmental knee arthroplasty (UKA) and anterior cruciate ligament reconstruction (ACLR). The authors hypothesized that the combined procedure leads to good long-term outcome in patients with isolated medial knee osteoarthritis (OA) and anterior cruciate ligament (ACL) deficiency.

**Methods:**

Twenty-three patients with ACL deficiency and concomitant medial knee OA were treated from 2008 to 2016 with a combined UKA (Oxford Partial Knee) and ACLR using a hamstring tendon autograft. The follow-up assessment included VAS pain score, Lysholm score, Oxford Knee Score (OKS), American Knee Society scores (AKSS), International Knee Documentation Committee (IKDC 2000), Tegner and UCLA activity scores. Instrumented laxity test was done using the KT-1000 arthrometer. Survivorship analysis was performed using the Kaplan–Meier method. Implant loosening and disease progression was assessed by conventional radiography.

**Results:**

Average follow-up duration was 10 years (6–14.5). VAS, Lysholm, Tegner and UCLA scores improved significantly. OKS, AKSS and IKDC 2000 showed excellent results on follow-up. Implant survivorship was 91.4% at 14.5 years. There were 2 revisions with conversion to total knee arthroplasty at 6 and 12 years postoperatively due to trauma and disease progression, respectively. There were no radiological or clinical signs of instability or disease progression in any of the remaining knees. The side-to-side difference using the KT-1000 arthrometer was insignificant.

**Conclusions:**

UKA combined with ACLR is an effective therapeutic option with good outcome and return to sport rate on the long-term.

**Level of evidence:**

IV.

## Introduction

The best treatment approach for medial knee osteoarthritis (OA) with existing anterior cruciate ligament (ACL) deficiency remains unclear. Primary OA progressively leads to secondary ACL instability as well as shortening of the medial collateral ligament (MCL) and gradual damage to the remaining knee compartments. In this patient group, a total knee arthroplasty (TKA) is indicated [22]. Patients with a primary ACL injury who develop unicompartmental OA are usually young and active. Chronic recurrence of giving way episodes in which posterior femoral subluxation occurs progressively leads to posteromedial OA by causing various degenerative alterations of the joint and accentuating the varus morphometry [24]. An ACL injury increases the risk of OA tenfold compared to the normal population [15].

Results of unicompartmental knee arthroplasty (UKA) have shown excellent long-term outcome with high patient satisfaction [13, 16]. According to the current consensus, UKA is indicated in patients suffering from isolated medial OA with intact cruciate and collateral ligaments [2]. ACL deficiency is considered an contraindication to UKA due to increased failure rates [7]. An in vitro robotic study showed that knee stability is not altered by a medial UKA but an ACL is essential to avoid pronounced anterior tibial translation [23].

The combined procedure of UKA and ACL reconstruction (ACLR) was proposed as a solution to this predicament and early results are encouraging [17, 25]. The ACLR seems to restore kinematics in the UKA knee in magnitudes similar to those in ACL-intact knees [6]. Several authors reported good clinical and radiological outcomes using the combined procedure with a mid-term follow-up duration [1, 11]. Long-term results are too scarce to develop a reliable statement [9, 10]. The aim of the study was to follow a consecutive group of patients who received a simultaneous ACLR and UKA. It was hypothesized that on long-term follow-up, the combined procedure will preserve knee stability and function with a survivorship equivalent to isolated UKA.

## Patients and methods

A consecutive group of 23 patients (18 male, 5 female) received a UKA (Oxford Partial Knee, Zimmer Biomet Inc., Warsaw, Indiana, USA) in combination with ACLR using the hamstring tendon autograft between 2008 and 2016. All patients suffered from a primary ACL rupture and concomitant Medial knee OA. Diagnosis of the OA was done using conventional radiographs including anteroposterior (AP) view, a lateral view and AP views with varus/valgus stress tests. The ACL injury was evaluated clinically (Lachman test) and confirmed by magnetic resonance imaging (MRI). The average age at the time of surgery was 48 years (44–69). A total 19 patients received a cemented UKA and 4 patients received a non-cemented UKA. The surgeries were performed in 3 institutions by 4 senior surgeons who were trained in the same institution and are experienced with ACLR and UKA.

All patients met the inclusion criteria: body mass index < 30, isolated medial OA, varus knee deformity < 10 degrees, flexion contracture < 5 degrees, absence of patellofemoral OA symptoms, and absence of previous knee surgery or other ligamentous insufficiency.

The following scores were assessed on follow-up using questionnaires: Visual Analog Score (VAS), Lysholm score, Oxford Knee Score (OKS), American Knee Society scores (AKSS), International Knee Documentation Committee (IKDC 2000), Tegner and UCLA activity levels [4, 20]. Laxity with AP translation was assessed clinically using the Lachman test with a knee flexion of 30°. Instrumented laxity test of the operated knee was measured in millimeters using the reliable KT-1000 (Medmetric, San Diego, CA, US) and compared to the contralateral knee [3, 21]. Side-to-side differences were calculated. Standard AP and lateral radiographs were done only in defined timeframes following the surgical intervention (6 weeks/6 months/1 year/3 years/5 years/10 years/15 years) and were analyzed for presence of implant loosening or disease progression to the lateral compartment.

The study was approved by the institutional review board of the University of Heidelberg (Registration code S-093). It was conducted in accordance with the Helsinki Declaration of 1975, as revised in 2013. All patients provided written informed consent for participation in the study.

### Surgical technique

The patient was positioned lying supine with a thigh tourniquet inflated to 280 mmhg. The knee was positioned hanging in a leg holder. A preliminary diagnostic arthroscopy was performed to confirm the indication of the combined procedure. A vertical incision over the pes anserinus was performed. The semitendinosus tendon was harvested with a tendon stripper and a four-stranded ACL replacement was prepared. The femoral canal was positioned and drilled through an anteromedial portal after assessing the diameter of the autograft. A medial mini-arthrotomy was then performed by extending the original incision proximally. Oxford UKA was then performed. The tibial resection was done few millimeters medial to the ACL stump to allow correct positioning of the tibial tunnel and avoid overlapping with the keel of the tibial component. The femoral resection was made in a standard manner for Oxford UKA. After insertion of the trial components, the tibial tunnel was made. It was positioned steeper and more anteriorly to avoid weakening the medial tibial plateau. Cementation was then done in select patients after covering the tibial tunnel. The original femoral and tibial implants were inserted and anchored. A sample of the tibial bearing was inserted to obtain the perfect ligament tensioning and balance. After polymerisation of the cement, the autograft was passed through the tunnels and fixated to the femur with a cortical button using the ACL Tightrope II® (Arthrex, FL, USA) and to the tibia with an interference absorbable screw and an additional suture disk. The definitive tibial bearing was inserted after retesting the knee range of motion (ROM).

### Postoperative rehabilitation protocol

The postoperative rehabilitation protocol was brace-free with focus on regaining active and passive ROM. Isometric muscle exercises were started on the day after the operation. For the first 6 weeks, walking with partial weight-bearing was allowed. Flexion was limited to 90° in the first 4 weeks while immediate regaining of full knee extension was encouraged. Starting from the 4th week, proprioception exercises were added including assisted single-leg balance and heel-to-toe walking. Bicycling was allowed 4 weeks postoperatively. Strength training with focus on regaining muscle mass was gradually started 6 weeks postoperatively. Jogging was allowed 3 months postoperatively. Noncontact sports were allowed after 6 months and contact sports were allowed 1 year following surgery.

### Statistical analysis

Data analysis was conducted using IBM SPSS Statistics version 25.0 (IBM Corp., Armonk, N.Y., USA). The mean and standard deviation were calculated. Wilcoxon test was used to compare the preoperative and follow-up scores. For all tests, *p* values of < 0.05 were considered to be significant. Kaplan–Meier analysis was used for survival analysis. A revision for any reason with or without removal/exchange of any part of the initial implant was used as an endpoint.

## Results

The average follow-up duration was 10 years (6–14.5). No patients were lost to follow-up. There were significant improvements in VAS, Lysholm, UCLA and Tegner scores on follow-up. Twenty-two patients achieved a good or excellent OKS result. In the IKDC 2000, 19 patients scored over 70. Table 1 displays all assessed scores.

**Table 1:** Preoperative and follow-up scores presented as mean ± standard deviation (range) with corresponding *p* values

Score	Preoperative value	Postoperative value	*p* value
Lysholm	45 ± 20.8 (17–77)	85.5 ± 20.9 (44–100)	0.005
Tegner	2.8 ± 1.75 (1–5)	3.6 ± 1.25 (0–7)	0.005
UCLA	5.1 ± 2 (2–7)	6.7 ± 1.5 (4–8)	0.0071
VAS	7.4 ± 1.95 (3–10)	1.3 ± 1.6 (0–5)	0.0001
OKS	–	40 ± 8.2 (29–48)	–
IKDC 2000	–	76.7 ± 35.5 (40–97)	–
AKSS-A	–	91.5 ± 11.6 (74–100)	–
AKSS-B	–	90 ± 15.85 (50–100)	–

*VAS* visual analog scale, *OKS* Oxford knee score, *IKDC* International Knee documentation committee, *AKSS* American Knee Society score

**Fig. 1:**
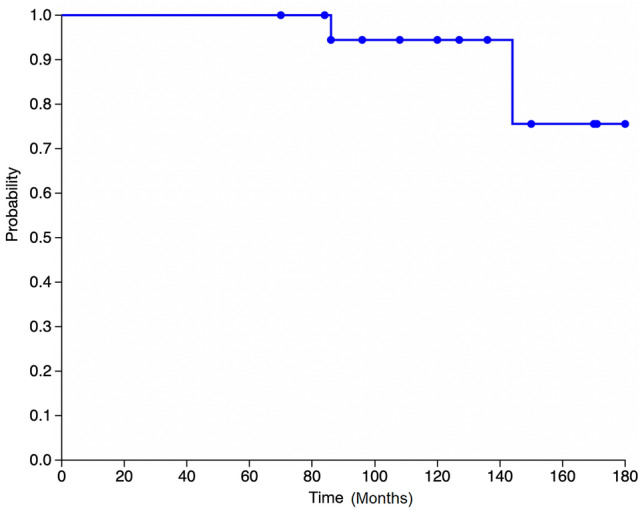
Kaplan-Meier curve showing revision for any reason with or without removal/exchange of any part of the initial implant as an endpoint (95% Confidence Interval [CI] 75.0–100.0)

There were two revisions (8.7%) occurring at a mean of 9 years, both of which were converted to TKA. One patient received a conversion procedure after incurring a trauma 6 years postoperatively and suffering from persistent pain and instability in the knee. The other patient received a conversion procedure 12 years postoperatively after developing symptomatic lateral knee OA.  The first patient reported being satisfied with the treatment before incurring the trauma. The implant survival rate was 100% at 5 years, 95,7% at 10 years and 91,4% at 14.5 years. Kaplan–Meier survival curve is displayed in Fig. 1. All patients were satisfied with the procedure and 22 out of 23 patients reported that they would undergo the same procedure again.

**Fig. 2:**
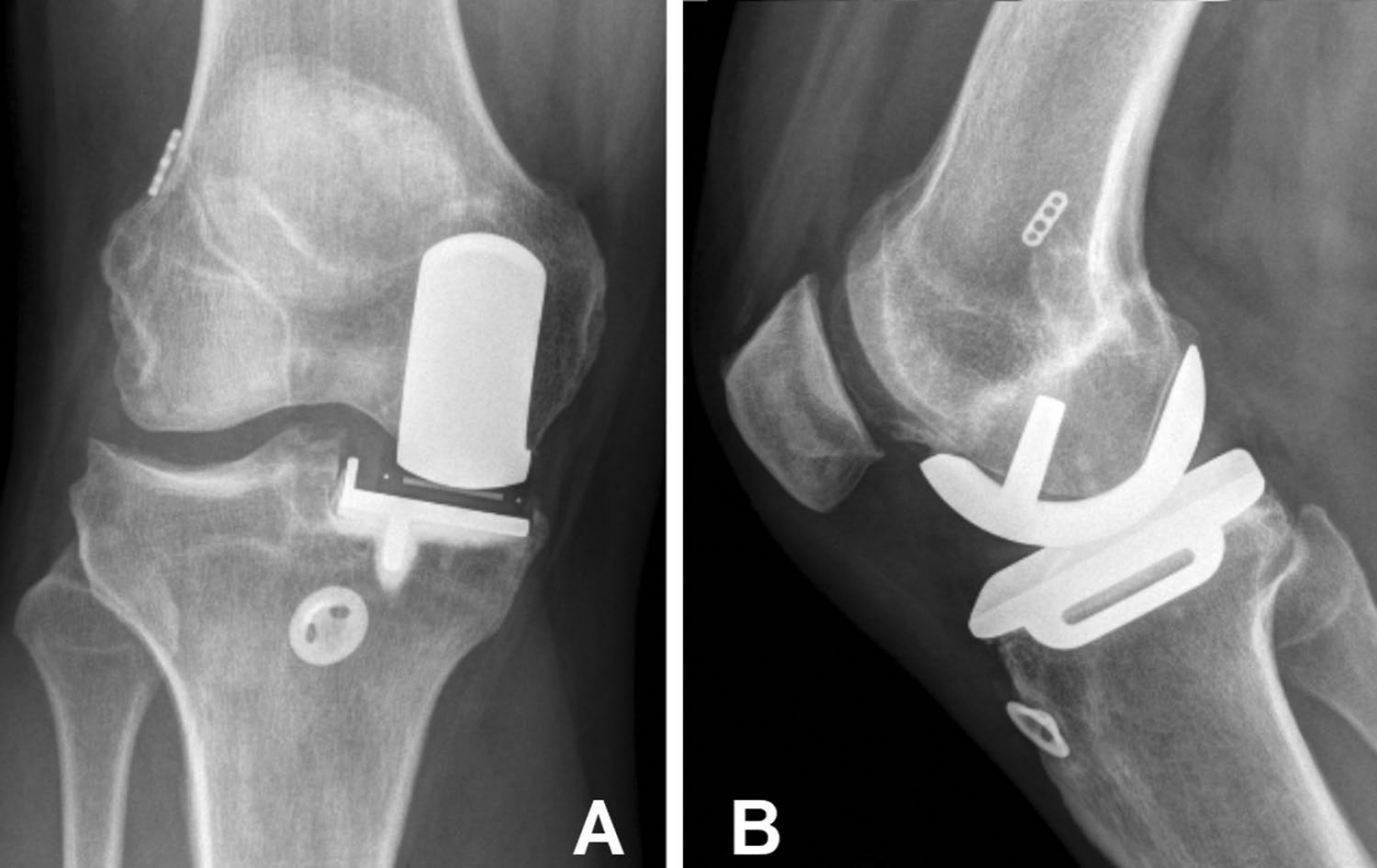
Conventional radiographs with anterioposterior (**A**) and lateral views (**B**) in a 54 year-old patient 10 years postoperatively after unicompartmental knee arthroplasty and anterior cruciate ligament reconstruction of the right knee

There was no clinical evidence of instability in any of the knees as evaluated with clinical and instrumental laxity testing. Full knee extension was reached by 21 patients and 2 patients had a 5° knee extension deficit. Knee flexion ranged between 105 and 140° with 21 patients having a knee flexion of more than 120°. The return to sport rate was 100%. All knees had a firm endpoint in the Lachman test. The AP-Translation using the KT-1000 Arthrometer measured 3.9 (Range 2–10) for the operated knee and 2.9 (2–6) for the contralateral knee (*p* value: n.s). The average side-to-side difference between the operated and unoperated knee was 1.8 (0–6) with 3 patients having a difference of 3 mm or more.  The radiological evaluation showed no signs of implant loosening or disease progression on follow-up except in 1 patient who recieved a conversion procedure 12 years postoperatively. Figure 2 displays the follow-up radiographs of a patient 10 years postoperatively.

## Discussion

The most important finding of the present study is that long-term function and implant survival following Oxford UKA combined with ACLR are excellent. In a recent comprehensive systemic review, Albo and colleagues concluded that the combined procedure is safe with a significant functional and clinical improvement [1]. Iriberri et al. published the results in 8 patients with a long-term follow-up and cited concerns over potential long-term deterioration after the 10 year mark since 2 out of 8 patients received a conversion to TKA due to disease progression [9]. In a large cohort described by Kennedy et al. which included patients receiving the combined procedure either simultaneously or separately, only 2 patients received a conversion procedure due to disease progression on the long-term [10]. In the present cohort, one patient received a TKA 12 years following the first operation due to primary disease progression and 1 patient received it 6 years postoperatively after trauma to the knee. Reports of the outcome of isolated UKA described implant survival rate of 80–95% on the long-term and only 30% of all revisions are related to disease progression [5, 19]. The cumulative results of the combined procedure are thus equivalent to those of isolated UKA [18]. The additional ACLR does not seem to increase the risk of disease progression.

Patients with ACL insufficiency secondary to OA are not good candidates for the combined procedure due to the presence of anteromedial tibial erosion with degeneration of other articular compartments and remaining ligamentous structures. If the ACL rupture is associated with a MCL rupture, intraoperative balancing is not possible because of medial instability. A chronic ACL rupture with preexisting medial OA leads to contracture of the MCL. Even then, correct balancing is no longer possible. If the MCL and lateral compartment are intact, balancing of the UKA in the case of ACL insufficiency or a recent rupture is unproblematic. The tensioned MCL determines the height of the bony resections. The integrity of the medial ligament must therefore be verified preoperatively. During balancing, the anterior translation needs to be neutralized by applying slight pressure from the ventral side on the proximal tibia. Moreover, before insertion of the ACL autograft after the UKA is carried out, a trial tibial bearing needs to be inserted to avoid intraarticular varus hyper-compression.

Simultaneous high tibial osteotomy (HTO) and ACLR in the setting of medial OA due to ACL deficiency is another treatment option for physically active young patients that has demonstrated good outcomes with return to recreational sports [8, 12, 14]. However, the literature collectively showed that HTO with ACLR leads to a higher complication rate than combined UKA and ACLR even though the survival rate is similar [14]. Combined UKA and ACLR additionally offers advantages in terms of faster recovery and better long-term functional outcome. The authors believe that HTO with ACLR can be an appropriate choice in select patients, especially those with an excessive posterior tibial slope and severe varus malalignment [8].

Limitations of the present study include the retrospective design and incomplete preoperative score assessment. The number of patients is limited and is distributed among 3 institutions, which dilutes the results. The surgeries were also done by 4 senior surgeons. However, they were all trained in one university hospital before going to neighboring hospitals and their surgical approach did not differ. The medical literature would benefit from prospective comparison studies with larger sample sizes and follow-up durations of more than 20 years.

## Conclusion

Simultaneous ACLR and UKA is a viable treatment option with a good long-term outcome and return to sport rate in patients with an ACL injury and isolated medial knee OA. This approach should be included in the treatment portfolio and patients’ long-term expectations can be raised. Proper surgical indication and execution remain crucial for the success of the procedure.

## Data Availability

The datasets used and/or analyzed during the current study are available from the corresponding author on reasonable request.

## Abbreviations

OA: Osteoarthritis
TKA: Total knee arthroplasty
UKA: Unicompartmental knee arthroplasty
ACL: Anterior cruciate ligament
ACLR: Anterior cruciate ligament reconstruction
AP: Anteroposterior
ROM: Range of motion
MRI: Magnetic resonance imaging
MCL: Medial collateral ligament
AKSS: American knee society scores
OKS: Oxford knee score
VAS: Visual analog scale
IKDC 2000: International knee documentation committee
